# COVID-19–Associated Pulmonary Aspergillosis, March–August 2020

**DOI:** 10.3201/eid2704.204895

**Published:** 2021-04

**Authors:** Jon Salmanton-García, Rosanne Sprute, Jannik Stemler, Michele Bartoletti, Damien Dupont, Maricela Valerio, Carolina Garcia-Vidal, Iker Falces-Romero, Marina Machado, Sofía de la Villa, Maria Schroeder, Irma Hoyo, Frank Hanses, Kennio Ferreira-Paim, Daniele Roberto Giacobbe, Jacques F. Meis, Jean-Pierre Gangneux, Azucena Rodríguez-Guardado, Spinello Antinori, Ertan Sal, Xhorxha Malaj, Danila Seidel, Oliver A. Cornely, Philipp Koehler, The FungiScope European Confederation of Medical Mycology/The International Society for Human and Animal Mycology Working Group

**Affiliations:** University of Cologne, Cologne, Germany (J. Salmanton-García, R. Sprute, J. Stemler, E. Sal, X. Malaj, D. Seidel, O.A. Cornely, P. Koehler);; L’Azienda Ospedaliero-Universitaria di Bologna Policlinico S. Orsola, Bologna, Italy (M. Bartoletti);; Alma Mater Studiorum University of Bologna, Bologna (M. Bartoletti);; Hospices Civils de Lyon, Lyon, France (D. Dupont);; Université Claude Bernard Lyon 1, Lyon (D. Dupont);; Centre de Recherche en Neurosciences de Lyon, Institut National de la Santé et de la Recherche Médicale, Centre National de la Recherche Scientifique, Lyon (D. Dupont);; Instituto de Investigación Sanitaria Gregorio Marañón, Madrid, Spain (M. Valerio, M. Machado, S. de la Villa);; Hospital Clinic, Institute of Biomedical Research August Pi i Sunyer, Barcelona, Spain (C. Garcia-Vidal);; Hospital Universitario La Paz, Madrid (I. Falces-Romero);; University Medical Center Hamburg-Eppendorf, Hamburg, Germany (M. Schroeder);; Centro Médico ABC, Mexico City, Mexico (I. Hoyo);; University Hospital Regensburg, Regensburg, Germany (F. Hanses);; Federal University of Triângulo Mineiro, Uberaba, Brazil (K. Ferreira-Paim);; Istituto di Ricovero e Cura a Carattere Scientifico San Martino Polyclinic Hospital, Genoa, Italy (D.R. Giacobbe);; Canisius Wilhelmina Hospital, Nijmegen, the Netherlands (J.F. Meis);; Federal University of Paraná, Curitiba, Brazil (J.F. Meis);; University of Rennes I, Institut National de la Santé et de la Recherche Médicale, École des Hautes Études en Santé Publique, Institut de Recherche en Santé, Environnement et Travail, Rennes, France (J.-P. Gangneux);; Hospital de Cabueñes, Gijón, Spain (A. Rodríguez-Guardado);; Instituto de Investigación Sanitaria del Principado de Asturias, Oviedo, Spain (A. Rodríguez-Guardado);; University of Milan, Milan, Italy (S. Antinori); German Centre for Infection Research, Cologne (O.A. Cornely)

**Keywords:** SARS-CoV-2, *Aspergillus*, voriconazole, intensive care unit, aspergillosis, SARS-CoV-2, COVID-19, respiratory infections, severe acute respiratory syndrome coronavirus 2, coronavirus disease, zoonoses, viruses, coronaviruses, fungi

## Abstract

Pneumonia caused by severe acute respiratory syndrome coronavirus 2 emerged in China at the end of 2019. Because of the severe immunomodulation and lymphocyte depletion caused by this virus and the subsequent administration of drugs directed at the immune system, we anticipated that patients might experience fungal superinfection. We collected data from 186 patients who had coronavirus disease–associated pulmonary aspergillosis (CAPA) worldwide during March–August 2020. Overall, 182 patients were admitted to the intensive care unit (ICU), including 180 with acute respiratory distress syndrome and 175 who received mechanical ventilation. CAPA was diagnosed a median of 10 days after coronavirus disease diagnosis. *Aspergillus fumigatus* was identified in 80.3% of patient cultures, 4 of which were azole-resistant. Most (52.7%) patients received voriconazole. In total, 52.2% of patients died; of the deaths, 33.0% were attributed to CAPA. We found that the cumulative incidence of CAPA in the ICU ranged from 1.0% to 39.1%.

Cases of pneumonia caused by severe acute respiratory syndrome coronavirus 2 (SARS-CoV-2) were first described in Wuhan, China, at the end of December 2019 (*1*). The infection rapidly spread, causing the coronavirus disease (COVID-19) pandemic (*2*).

Because SARS-CoV-2 and treatments such as dexamethasone or tocilizumab can impair the immune system, some researchers anticipated the possibility of fungal superinfection among COVID-19 patients (*3*–*6*). As of August 2020, researchers have documented COVID-19–associated pulmonary aspergillosis (CAPA) (*7*–*9*), invasive candidiasis (*10*), coccidioidomycosis (*11*), fusariosis (*12*), histoplasmosis (*13*), mucormycosis (*14*), pneumocystosis (*15*), and saccharomycosis (*16*). Varying cumulative rates of CAPA have been described, including rates of 0.7%–7.7% among COVID-19 patients (*17*,*18*), 2.5%–39.1% among ICU patients with COVID-19 (*19*,*20*), and 3.2%–29.6% among COVID-19 patients on mechanical ventilation (*7*,*17*). Many of these patients lack the concurrent conditions usually associated with invasive pulmonary aspergillosis (IPA) such as malignancies, neutropenia, or history of allogeneic stem cell or solid organ transplantation (*21*). Admission to the ICU or severe influenza are also risk factors for IPA in nonneutropenic patients (*22**–**25*). Reports of CAPA have been mostly limited to a few single-center studies; therefore, a comprehensive analysis of international distribution currently is lacking (*4*).

We analyzed reports in the literature (*26**–**50*; references *51*–*54*in Appendix) and the FungiScope registry (reference *55* in Appendix) to describe baseline conditions, clinical management, and associated deaths in CAPA patients. This analysis also contextualizes the available cumulative incidences.

## Methods

**Figure 1:**
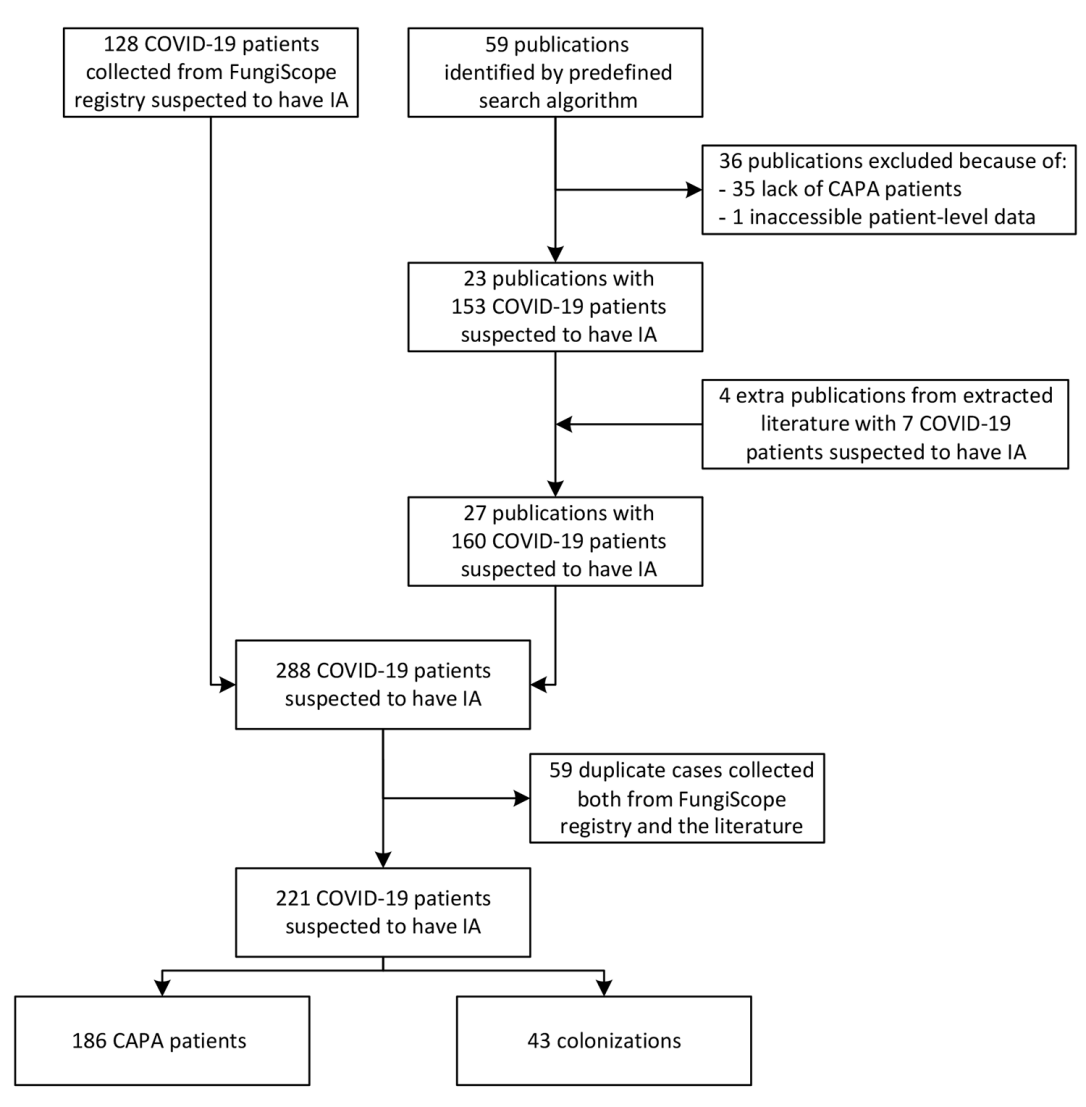
Enrollment process in study of patients with CAPA, March–August 2020. Patients were identified in the FungiScope registry and academic literature using the search string “(Aspergill*) AND (invasive OR putative OR probable OR infection OR case OR patient OR report) AND (COVID* OR corona* OR SARS-CoV-2) (Appendix Table 1). The initial 288 COVID-19 patients suspected to have IA were revised in a deduplication process; 59 double entries were identified. Only 1 report per patient was maintained. Thus, 221 individual COVID-19 patients suspected to have IA were assessed for CAPA. CAPA, COVID-19–associated pulmonary aspergillosis; COVID-19, coronavirus disease; EORTC/MSG, European Organization for Research and Treatment of Cancer/Mycoses Study Group; IA, invasive aspergillosis.

We conducted a retrospective analysis using clinical data of patients worldwide who received a CAPA diagnosis during March 1–August 31, 2020. Our analysis comprised data from the FungiScope registry and academic literature (Figure 1). 

FungiScope (https://www.clinicaltrials.gov; National Clinical Trials identifier NCT01731353) is a global registry for emerging invasive fungal infections. FungiScope was approved by the local ethics committee of the University of Cologne, Cologne, Germany (study ID 05-102). The registry includes patients with invasive aspergillosis since 2019. FungiScope’s methods have been described previously (reference *55* in Appendix).

In addition, we conducted a literature search using the PubMed database (https://pubmed.ncbi.nlm.nih.gov) for suspected CAPA cases occurring in March–August 2020. We used the search string “(Aspergill*) AND (invasive OR putative OR probable OR infection OR case OR patient OR report) AND (COVID* OR corona* OR SARS-CoV-2),” which identified 59 published articles. We reviewed and extracted relevant data from each of the publications. When necessary, we contacted authors for additional details (Appendix).

We reviewed each patient report using multiple diagnostic definitions. First, we evaluated the patients according to the consensus definition of Koehler et al. (reference *56* in Appendix); we classified the patients as having proven, probable, or possible CAPA. We used alternative definitions to evaluate patients who were nonclassifiable because of lack of essential information, such as the volume of saline recovered by nondirected bronchial lavage (NBL) fluid applied. We categorized the nonclassifiable patients as proven or probable according to the European Organization for Research and Treatment of Cancer/Invasive Fungal Infections Cooperative Group and the National Institute of Allergy and Infectious Diseases Mycoses Study Group criteria for invasive fungal infections (*21*) or as proven, putative, and colonized according to the AspICU algorithm for IPA in critically ill ICU patients by Blot et al. (*23*). We considered severe COVID-19 with acute respiratory distress syndrome (ARDS) to be a valid host criterion (i.e., acquired immunodeficiency) (*8*). We considered patients who met >1 definition to have CAPA; we categorized the rest as nonclassifiable.

We collected data on patients’ demographic characteristics and baseline conditions. We also collected data on abnormal radiographic images, mycologic evidence, signs and symptoms at CAPA diagnosis, site of infection, antifungal susceptibility testing, antifungal treatment, death at 6 and 12 weeks after CAPA diagnosis, and absolute death. In addition, we calculated the length of time between COVID-19 and CAPA diagnoses, CAPA diagnosis and most recent healthcare contact with the patient, ICU admission and CAPA diagnosis, and installation of mechanical ventilation and CAPA diagnosis. The contribution of CAPA to patient death (i.e., attributable mortality) was assessed by the treating medical team (Appendix Table 2). To determine the cumulative incidence of CAPA in the facilities included in the analysis, we asked each institution for 3 different denominators: the total number of COVID-19 patients, the number of COVID-19 patients admitted to the ICU, and the number of COVID-19 patients admitted to the ICU who needed mechanical ventilation during March–August 2020.

### Statistical Analysis

We did not calculate an a priori sample size for this exploratory study. To analyze the demographic and clinical characteristics of patients with CAPA, we describe categorical variables using frequencies and percentages; we describe continuous variables using medians and interquartile ranges (IQRs). We used SPSS Statistics 25.0 (IBM, https://www.ibm.com) for statistical analyses.

## Results

**Figure 2:**
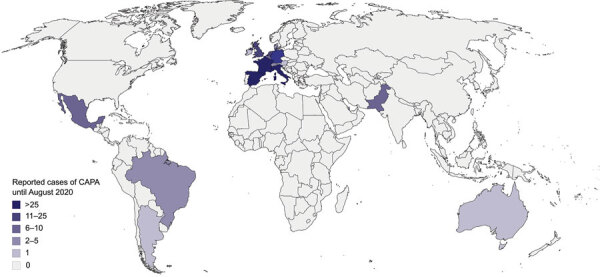
Global distribution of the 186 CAPA patients reported in the literature and FungiScope registry, March–August 2020. In total, 39 patients were from France, 36 from Italy, 26 from Spain, 23 from Germany, 14 from the Netherlands, 11 from the United Kingdom, 9 from Pakistan, 8 from Belgium, 6 from Mexico, 3 from Brazil, 3 from Switzerland, 2 from Denmark, 2 from Qatar, 1 from Argentina, 1 from Australia, 1 from Austria, and 1 from Ireland (Appendix Table 8). CAPA, COVID-19–associated pulmonary aspergillosis; COVID-19, coronavirus disease.

We identified 186 CAPA cases during March 1–August 31, 2020, in 17 different countries, according to European Organization for Research and Treatment of Cancer/Invasive Fungal Infections Cooperative Group and the National Institute of Allergy and Infectious Diseases Mycoses Study Group criteria (*21*), Blot et al. algorithm (*23*), and Koehler et al. consensus definition (reference *56* in Appendix) (Figures 1, 2; Appendix Table 1). We identified 62 (33.3%) cases from literature, 45 (24.2%) from the FungiScope registry, and an additional 79 (42.5%) in both sources (Table 1). The median age among persons with CAPA was 68 years (IQR 59–73 years; range 15–87 years). Most (135; 72.6%) patients were men (Table 2).

**Table 1:** Pathogens of 186 patients with coronavirus disease–associated pulmonary aspergillosis, March–August 2020*

Characteristic	No. (%)
Pathogens†	
* Aspergillus fumigatus*	122 (65.6)
* A. niger*	13 (7.0)
* A. flavus*	10 (5.4)
* A. terreus*	6 (3.2)
* A. calidoustus*	1 (0.5)
* A. lentulus*	1 (0.5)
* A. nidulans*	1 (0.5)
* A. penicillioides*	1 (0.5)
* A. versicolor*	1 (0.5)
* A. tubingensis*	1 (0.5)
*Aspergillus* spp. (culture)‡	1 (0.5)
*Aspergillus* spp. (serologic techniques)	34 (18.3)
Other pathogens§	40 (21.5)
Case definition	
EORTC/MSG criteria (*21*)	
Proven	7 (3.8)
Probable	10 (5.4)
Nonclassifiable	169 (90.9)
AspICU algorithm (*23*)¶	
Proven	7 (3.8)
Putative	142 (76.3)
Colonization	34 (18.3)
Nonclassifiable	3 (1.6)
Consensus definition (reference *57* in Appendix)	
Proven	7 (3.8)
Probable	82 (44.1)
Possible	19 (10.2)
Nonclassifiable¶#	78 (41.9)
Mycologic evidence	
Culture**	152 (81.7)
Microscopy††	3 (1.6)
Histologic techniques‡‡	7 (3.8)
PCR§§	43 (23.1)
Galactomannan test¶¶	113 (60.8)

*Some patients had >1 pathogen or form of mycologic evidence. BAL, bronchoalveolar lavage; EORTC/MSG, European Organization for Research and Treatment of Cancer/Mycoses Study Group (*21*). †A total of 2 patients had *A. fumigatus* and *A. niger* coinfection, 1 patient had *A. flavus* and *A. fumigatus* coninfection, 1 patient had *A. flavus* and *A. niger* coinfection, 1 patient had *A. fumigatus* and *A. terreus* coinfection, and 1 patient had *A. fumigatus* and *A. versicolor* coinfection. ‡One patient had an *Aspergillus* spp. infection diagnosed by culture. No species determination was provided. Other patient samples were diagnosed as *Aspergillus* spp, using serologic techniques. §Small numbers of other pathogens were also retrieved from patient samples (Appendix Table 6). ¶AspICU method uses algorithm described by Blot et al. (*23*) for determining proven or putative aspergillosis in patients with influenza. #Up to 78 cases (41.9%) were considered nonclassifiable according to the definition (reference *56* in Appendix) because of lack of specific details about the type of aspiration performed. Of these, 75 (96.2%) were classified as putative according to the Blot et al. algorithm (*23*) and 3 (3.8%) as probable according to EORTC/MSG criteria (*21*). **Culture was used to analyze 50 BAL, 47 tracheal aspirate, 34 bronchial aspirate, 17 nondirected bronchial lavage, 3 sputum, 2 nonspecified lower respiratory tract, and 1 BAL and tracheal aspirate sample. ††Microscopy was used to analyze 1 BAL, 1 bronchial aspirate, and 1 tracheal aspirate sample. ‡‡Histologic techniques were used to analyze 7 lung tissue samples. §§PCR was used to analyze 16 BAL, 12 tracheal aspirate, 10 nondirected bronchial lavage, 3 bronchial aspirate, 1 lung tissue, and 1 serum sample. ¶¶Galactomannan tests were used to analyze 63 BAL, 30 serum or plasma, 22 nondirected bronchial lavage, 9 tracheal aspirate, 3 bronchial aspirate, and 1 sputum sample.

**Table 2:** Characteristics of 186 patients with coronavirus disease–associated pulmonary aspergillosis, March–August 2020*

Patient characteristic	No. (%)
Sex	
F	51 (27.4)
M	135 (72.6)
Median age, y (IQR)	68 (58–73)
COVID-19†	186 (100.0)
Median length of treatment, d (IQR)	7 (6–11)
Median time from COVID-19 diagnosis to CAPA, d (IQR)	10 (5–16)
Intensive care unit stay	182 (97.8)
Median length of stay before CAPA diagnosis, d (IQR)	8 (3–14)
Acute respiratory distress syndrome	180 (96.8)
Mechanical ventilation	175 (94.1)
Median time on ventilation before CAPA diagnosis, d (IQR)	7 (3–13)
Corticosteroid use	98 (52.7)
Concurrent conditions	
Chronic cardiovascular disease	94 (50.5)
Renal failure‡	74 (39.8)
Diabetes mellitus	64 (34.4)
Obesity	47 (25.3)
Chronic pulmonary disease	40 (21.5)
Hematologic or oncologic disease§	21 (11.3)
Hematologic malignancy	10 (5.4)
Solid tumor	9 (4.8)
Hematologic disease	2 (1.1)
Solid organ transplantation¶	4 (2.2)
Neutropenia	2 (1.1)
Other baseline conditions and characteristics#	70 (37.6)
Lung infection	186 (100.0)
Image abnormalities of the lungs	182 (97.8)
Computed tomography scan	134 (72.0)
Radiograph	88 (47.3)
Antifungal treatment	137 (73.7)
Median length of treatment, d (IQR)	16 (10–33)
Amphotericin B	36 (19.4)
Liposomal	23 (12.4)
Deoxycholate	11 (5.9)
Lipid complex	2 (1.1)
Echinocandins	24 (12.9)
Anidulafungin	10 (5.4)
Caspofungin	13 (7.0)
Micafungin	1 (0.5)
Ibrexafungerp	1 (0.5)
Triazoles	117 (62.9)
Voriconazole	98 (52.7)
Isavuconazole	23 (12.4)
Posaconazole	4 (2.2)
Fluconazole	1 (0.5)
Overall mortality	97 (52.2)
<6 wks	89 (47.8)
<12 wks	93 (50.0)
Median time to death, d (IQR)	9 (3–18)
Cause of death**	
CAPA	32 (17.2)
COVID-19	51 (27.4)
Other	36 (19.4)
Median length of observation from CAPA diagnosis, d (IQR)	22 (7–42)

*Values are no. (%), except as indicated. Some patients had >1 baseline condition or characteristic, image abnormality, or antifungal drug. CAPA, COVID-19–associated pulmonary aspergillosis; COVID-19, coronavirus disease. †By definition, all CAPA patients had COVID-19 (Appendix Table 3). ‡In total, 54 patients had acute renal failure, 18 had chronic renal failure, and 2 had nonspecified renal failure. §In total, 9 patients had hematologic malignancy: 3 had chronic leukemia, 3 had lymphoma, 2 had myelodysplastic syndrome, and 1 had acute leukemia. Eight patients had a solid tumor: 1 had breast cancer, 1 had carcinoma, 1 had cervical/uterine cancer, 1 had lung cancer, 1 had esophageal carcinoma, 1 had prostate cancer, 1 had testicular cancer, and 1 had urothelial carcinoma. Two patients had hematologic disease: 1 had acquired hemophilia type A and 1 had hemophagocytic lymphohistiocytosis. ¶In total, 3 patients had a kidney transplant, 1 had a liver transplant, and 1 had a lung transplant. #Small numbers of patients had other concurrent conditions and characteristics (Appendix Table 7). **In total, 32 patients died of CAPA or CAPA/COVID-19: 7 died of CAPA only; 25 died of CAPA and COVID-19. In addition, 26 died of COVID-19 only.

Nearly all (182; 97.8%) patients were admitted to the ICU, most for ARDS (180; 96.8%) or mechanical ventilation (175; 94.1%). Other common baseline conditions and characteristics included corticosteroid administration (98; 52.7%), chronic cardiovascular disease (94; 50.5%), renal failure (74; 39.8%), diabetes mellitus (64; 34.4%), and obesity (47; 25.3%). Overall, 40 (21.5%) patients had chronic pulmonary disease (Table 2).

In total, 110 (59.1%) patients received either hydroxychloroquine (98; 52.7%) or chloroquine (12; 6.5%) for treatment of COVID-19. Sixty-eight (36.6%) patients received corticosteroids, mainly methylprednisolone monotherapy (26; 14.0%) or antivirals (67; 36.0%), especially lopinavir/ritonavir monotherapy (56; 30.1%). COVID-19 treatment had a median duration of 7 days before recovery or death (IQR 6–11 days; range 1–32 days) (Table 2; Appendix Table 3).

In 152 (81.7%) patients, CAPA was diagnosed a median of 10 days (IQR 5–16 days; range 0–51 days) after a positive respiratory sample for SARS-CoV-2 infection by reverse transcription PCR. Among all patients, *Aspergillus fumigatus* was the most frequently reported (122/152; 65.6%) pathogen. Six patients (3.2%) had cultures positive for >1 *Aspergillus* species. Samples mainly were from bronchoalveolar lavage (BAL) (50; 26.9%), tracheal aspirates (48; 25.8%), or bronchial aspirates (34; 18.3%). In 55 (29.6%) patients, culture was the only diagnostic tool that produced a positive result. Galactomannan (GM) levels were positive (i.e., optical density index ≥1.0) in samples from 113 (60.8%) patients, including BAL samples from 63 (33.9%) patients, serum or plasma from 29 (15.6%), and NBL from 22 (11.8%). Histologic techniques were used for diagnosis in 7 (3.8%) cases. Abnormal radiographic imaging was found in 182 (97.8%) patients, either in computed tomography scans (94; 50.5%), in chest radiographs (48; 25.8%), or both (40; 21.5%) (Table 2).

Overall, 30 (16.1%) patients provided samples for >1 antifungal susceptibility test, such as microdilution according to European Committee on Antimicrobial Susceptibility Testing guidelines (20; 10.8%) (reference *57* in Appendix), Etest (11; 5.9%), and Clinical and Laboratory Standards Institute microdilution procedures (1; 0.5%) (reference *58* in Appendix). The tests were predominantly performed on *A. fumigatus* (29; 15.6%) isolates, 3 of which had the TR34L98H resistance mutation in the *cyp51A* gene. One (0.5%) patient had voriconazole-resistant *A. lentulus* (MIC 2 µg/mL by EUCAST guidelines) (Appendix Table 4).

Of 186 CAPA patients, 49 (26.3%) patients did not receive mold-active antifungal therapy. The most common treatments were triazoles (117; 62.9%), especially voriconazole (98; 52.7%, including 79 patients for whom voriconazole was a first-line treatment) and isavuconazole (23; 12.4%). In total, 34 (19.4%) patients received amphotericin B, especially liposomal amphotericin B (23; 12.4%). Of the patients who received amphotericin B, 15 (65.2%) received it as first-line treatment. Antifungal treatment was administered for a median of 16 days before recovery or death (IQR 10–33 days; range 1–92 days) (Table 2; Appendix Table 5).

In total, 97 (52.2%) patients died, most (89; 47.8%) <6 weeks after CAPA diagnosis. In 32 (17.2%) patients, death was attributed to *Aspergillus*; including 25 (13.4%) patients who died of aspergillosis and COVID-19 infection. Patients were observed for a median of 22 days (IQR 7–42 days; range 0–144 days) after CAPA diagnosis; survivors were treated for a median of 40 days (IQR 28–50 days; range 1–144 days) and patients who died for a median of 9 days (IQR 3–18 days; range 0–144 days) (Table 2).

In total, 19 of 39 institutions provided denominators for cumulative incidence over the duration of the study period. The CAPA incidence among all COVID-19 patients ranged from 0.1%–9.7%. Among COVID-19 patients admitted to ICU, cumulative incidences ranged from 1.0%–39.1%. Among patients admitted to ICU who needed mechanical ventilation, cumulative incidences ranged from 1.1%–47.4% (Table 3).

**Table 3:** Cumulative incidences of CAPA in 19 facilities, March–August 2020*

Country, site no.	CAPA cases, no.	Denominator, no. (% CAPA)			Timeframe
		COVID-19 patients	COVID-19 patients in ICU	COVID-19 patients on mechanical ventilation	
Argentina, I	2	673 (0.3)	163 (1.2)	69 (2.9)	Mar–Aug
Belgium, I	4	274 (1.5)	46 (8.7)	32 (12.5)	Mar–Aug
Belgium, II	4	NA	34 (11.8)	20 (20.0)	Mar–Apr
France, I	2	519 (0.4)	113 (1.8)	45 (4.4)	Mar–Aug
Germany, I	1	83 (1.2)	18 (5.6)	15 (6.7)	Mar–Aug
Germany, II	11	231 (4.8)	64 (17.2)	56 (19.6)	Mar–Aug
Germany, III	9	93 (9.7)	38 (23.7)	27 (33.3)	Mar–Aug
Germany, IV	7	123 (5.7)	76 (9.2)	57 (12.3)	Mar–Aug
Ireland, I	3	181 (1.7)	15 (20.0)	14 (21.4)	Mar–Aug
Italy, I	2	1,279 (0.2)	196 (1.0)	188 (1.1)	Mar–Aug
Italy, II	8	1,055 (0.8)	144 (5.6)	142 (5.6)	Mar–Aug
Mexico, I	6	312 (1.9)	131 (4.6)	115 (5.2)	Mar–Aug
Netherlands, I	9	NA	NA	53 (17.0)	Apr
Netherlands, II	6	483 (1.2)	118 (5.1)	NA	Mar–Aug
Pakistan, I	9	147 (6.1)	23 (39.1)	19 (47.4)	Mar–Apr
Spain, I	8	1,543 (0.5)	348 (2.3)	146 (5.5)	Mar–Aug
Spain, II	8	7,880 (0.1)	NA	NA	Mar–Aug
Spain, III	10	5,890 (0.2)	NA	NA	Mar–Aug
Switzerland, I	3	NA	118 (2.5)	80 (3.8)	Mar–May
United Kingdom, I	19	14,615 (0.1)	257 (7.4)	200 (9.5)	Mar–May
Total	131	35,381 (0.4)	1,902 (6.9)	1,278 (10.3)	Mar–Aug

*CAPA, COVID-19–associated pulmonary aspergillosis; COVID-19, coronavirus disease; ICU, intensive care unit; NA, not available.

## Discussion

We described 62 CAPA cases in the literature, 45 in the FungiScope registry, and 79 in both that were diagnosed during March 1–August 31, 2020. Men had a higher (2.6:1) prevalence of CAPA than women. This finding corresponds with a meta-analysis of >3 million COVID-19 patients that showed that men were at increased risk for severe COVID-19 and therefore complications such as CAPA (reference *59* in Appendix).

Most (97.8%) patients were admitted to the ICU, mainly because of ARDS, need for mechanical ventilation, or both. We found that corticosteroid administration, chronic cardiovascular disease, renal failure, diabetes mellitus, and obesity were common characteristics among these patients. Approximately 1 in 5 patients had chronic pulmonary disease. Patients had many similarities to influenza-associated pulmonary aspergillosis (IAPA) patients from Schauwvlieghe et al. (*22*), including similar rates of mechanical ventilation (IAPA 90.0% vs. CAPA 94.1%), corticosteroid administration (IAPA 56.0% vs. CAPA 52.7%), baseline renal failure (IAPA 42.0% vs. CAPA 39.8%), obesity (IAPA 30.0% vs. CAPA 25.3%), and chronic pulmonary disease (IAPA 16.0% vs. CAPA 21.5%). IAPA patients had a higher proportion of malignancies (30.0% vs. 11.3%) and solid organ transplantation (13.0% vs. 2.7%); however, CAPA patients had a higher prevalence of diabetes mellitus (12.0% vs. 34.4%). In our study, 50.5% of patients had chronic cardiovascular disease. These differences in the distribution of baseline characteristics between IAPA and CAPA patients reflects the epidemiology of COVID-19, which is more common among those with chronic cardiovascular disease, whereas hematologic or oncologic malignancies (*22*) are more common among those with IAPA (reference *60* in Appendix). Only 2% of COVID-19 patients have cancer (reference *61* in Appendix).

Available guidelines for aspergillosis management recommend diagnostic procedures such as respiratory culture and galactomannan index of BAL samples (references *60*,*62* in Appendix). However, these procedures have a high risk for aerosolization; safety precautions should be used when handling samples from COVID-19 patients (references *63*,*64* in Appendix). The elevated risk for SARS-CoV-2 transmission and the initial recommendation against using bronchoscopy for COVID-19 diagnosis (references *63*,*64* in Appendix) might explain the low number of BAL tests used to diagnose CAPA in our study. Schauwvlieghe et al. (*22*) diagnosed IAPA by using BAL cultures in 63.0% of the patients and the galactomannan test in 88.0%. In the current study, BAL cultures tested positive for *Aspergillus* in 26.9% of COVID-19 patients; galactomannan tests were positive in 33.9% of patients. Alternative respiratory sample sources (e.g., bronchial aspirate, NBL, tracheal aspirate, and sputum) were used for cultures in 35.4% of IAPA patients (*22*) and 31.2% of CAPA patients. Alternative samples also were used for galactomannan tests in 17.2% of CAPA patients; if optical density index cutoff values were not standardized for alternative samples, clinicians used the values for BAL. Almost all (97.8%) patients had imaging abnormalities; however, many had only marginally typical features of aspergillosis, hampering the differential diagnosis of CAPA according to radiologic criteria.

Positive isolates were recovered from 81.7% of CAPA patients. Similar to IAPA patients, the most common (80.3%) pathogen was *A. fumigatus* (*22*). In total, 5 patients had azole-resistant infections: 4 *A. fumigatus* and 1 *A. lentulus* infection. We noted 2 patients who had a possible previous exposure to triazoles. The professions of these 2 patients involved exposure to fungicides and manipulated organic matter containing triazole-resistant *A. fumigatus*. Therefore, the treating teams hypothesized that workplace exposure might have contributed to these patients’ illness. We found a similar proportion of patients with previous azole exposure as Verweij et al. (reference *65* in Appendix); however, the proportion found by Verweij et al. should be considered with caution because of small sample size.

Triazoles, especially voriconazole, were the most frequently administered antifungal drugs: 52.7% of the study cohort and 71.5% of the patients on antifungal treatment received voriconazole. We found that voriconazole use was associated with decreased death. The first-line use of voriconazole in 79 (80.6%) of 98 patients aligns with current recommendations (references *56*,*60*,*62* in Appendix).

We found a 50% mortality rate at 12 weeks after CAPA diagnosis. This finding is similar to the 51.0% mortality rate of IAPA patients in the same timeframe; however, these rates are almost 20 points higher than in other cohorts, such as aspergillosis patients with acute leukemia (33.8%) (reference *66* in Appendix). Nonetheless, in our study CAPA was attributed as the main reason for death in only 17.2% of the patients, whereas in Koehler et al. (reference *66* in Appendix), it was the main cause of death for 26.9% of patients with hematologic conditions.

We found an overall 6.9% cumulative incidence for CAPA among patients during the study period, although incidences varied by institution (1.0%–39.1% of CAPA patients admitted to ICU). In most facilities, the rates of CAPA were lower than those of IAPA (14%–19%) (reference *67* in Appendix). However, these ranges might vary according to diagnostic protocols in the different countries and healthcare facilities. Differences in screening practices for CAPA in COVID-19 patients might have affected detection rates and therefore our calculations of cumulative incidence (*8*). Further analyses are necessary to establish the geographic variance of this rate.

The first limitation of this study is that, because of the cross-sectional design of this study, we could not control for disease severity. Second, samples from the lower respiratory tract are the best way to differentiate between colonization and infection, but a low percentage of patients in this study had mycologic evidence from BAL culture or galactomannan tests. Third, we analyzed many cases from literature and could not contact certain authors for further details. In addition, institutions might not have documented all CAPA cases in the literature or FungiScope registry. Given the regional variability of the patient distribution, longitudinal studies might be a more appropriate tool to determine rates. Finally, because of the retrospective nature of the study, we could not retrieve the necessary clinical and diagnostic details of all patients. As a result, many patients were not classifiable according to the definitions used in this article, possibly contributing to an underdiagnosis of CAPA.

In conclusion, we described a large cohort of CAPA patients using cases from the literature and the FungiScope registry. CAPA occurs mostly in ICU patients on mechanical ventilation. We found that CAPA patients had high rates of chronic cardiovascular disease, renal failure, diabetes mellitus, and corticosteroid use. We also found that CAPA substantially contributed to a high death rate in COVID-19 patients, although cumulative incidence varied by treatment site. We believe that improved screening can identify and enable early treatment of CAPA.

Appendix Additional information on COVID-19–associated pulmonary aspergillosis.
