# Supplementary material for: COVID-19–Associated Pulmonary Aspergillosis, March–August 2020
Source: Emerg Infect Dis. 2021 Apr;27(4):1077–86. doi: 10.3201/eid2704.204895 (PMC8007287; doi:10.3201/eid2704.204895)
Supplement: Appendix — Additional information on COVID-19–associated pulmonary aspergillosis. [file 20-4895-Techapp-s1.pdf]

Article DOI: https://doi.org/10.3201/eid2704.204895

COVID-19–Associated Pulmonary Aspergillosis, March–August 2020

Appendix

Additional References

51. Falces-Romero I, Ruiz-Bastián M, Díaz-Pollán B, Maseda E, García-Rodríguez J; SARS-CoV-2 Working Group. Isolation of *Aspergillus* spp. in respiratory samples of patients with COVID-19 in a Spanish tertiary care hospital. Mycoses. 2020;63:1144–8. PubMed https://doi.org/10.1111/myc.13155

52. Machado M, Valerio M, Álvarez-Uría A, Olmedo M, Veintimilla C, Padilla B, et al.; COVID-19 Study Group. Invasive pulmonary aspergillosis in the COVID-19 era: an expected new entity. Mycoses. 2021;64:132–43. PubMed https://doi.org/10.1111/myc.13213

53. Marr KA, Platt A, Tornheim JA, Zhang SX, Datta K, Cardozo C, et al. Aspergillosis complicating severe coronavirus disease. Emerg Infect Dis. 2021;27:18–25. PubMed https://doi.org/10.3201/eid2701.202896

54. Lamoth F, Glampedakis E, Boillat-Blanco N, Oddo M, Pagani JL. Incidence of invasive pulmonary aspergillosis among critically ill COVID-19 patients. Clin Microbiol Infect. 2020;26:1706–8. PubMed https://doi.org/10.1016/j.cmi.2020.07.010

55. White PL, Dhillon R, Cordey A, Hughes H, Faggian F, Soni S, et al. A national strategy to diagnose COVID-19 associated invasive fungal disease in the intensive care unit. Clin Infect Dis. 2020;ciaa1298. PubMed https://doi.org/10.1093/cid/ciaa1298

56. Seidel D, Durán Graeff LA, Vehreschild MJGT, Wisplinghoff H, Ziegler M, Vehreschild JJ, et al.; FungiScope Group. FungiScope—global emerging fungal infection registry. Mycoses. 2017;60:508–16. PubMed https://doi.org/10.1111/myc.12631

57. Koehler P, Bassetti M, Chakrabarti A, Chen SCA, Colombo AL, Hoenigl M, et al.; European Confederation of Medical Mycology; International Society for Human Animal Mycology; Asia Fungal Working Group; INFOCUS Latin American Clinical Mycology/International Society for Human and Animal Mycology Working Group; International Society for Human and Animal Mycology Pan Africa Mycology Working Group; European Society for Clinical Microbiology; Infectious Diseases Fungal Infection Study Group; ESCMID Study Group for Infections in Critically Ill Patients; Interregional Association of Clinical Microbiology and Antimicrobial Chemotherapy; Medical Mycology Society of Nigeria; Medical Mycology Society of China Medicine Education Association; Infectious Diseases Working Party of the German Society for Haematology and Medical Oncology; Association of Medical Microbiology; Infectious Disease Canada. Defining and managing COVID-19–associated pulmonary aspergillosis: the 2020 ECMM/ISHAM consensus criteria for research and clinical guidance. Lancet Infect Dis. 2020 Dec 14 [Epub ahead of print]. PubMed

58. European Society of Clinical Microbiology and Infectious Disease. Clinical breakpoints for fungi (*Candida* and *Aspergillus* species). 2021 [cited 2020 Jun 1]. https://www.eucast.org/astoffungi/clinicalbreakpointsforantifungals

59. Clinical and Laboratory Standards Institute. Performance standards for antimicrobial susceptibility testing of filamentous fungi. 2017 [cited 2020 Jun 2]. https://clsi.org/media/1896/m61ed1_sample.pdf

60. Peckham H, de Gruijter NM, Raine C, Radziszewska A, Ciurtin C, Wedderburn LR, et al. Male sex identified by global COVID-19 meta-analysis as a risk factor for death and ITU admission. Nat Commun. 2020;11:6317. PubMed https://doi.org/10.1038/s41467-020-19741-6

61. Ullmann AJ, Aguado JM, Arikan-Akdagli S, Denning DW, Groll AH, Lagrou K, et al. Diagnosis and management of *Aspergillus* diseases: executive summary of the 2017 ESCMID-ECMM-ERS guideline. Clin Microbiol Infect. 2018;24:e1–38. PubMed https://doi.org/10.1016/j.cmi.2018.01.002

62. Desai A, Sachdeva S, Parekh T, Desai R. COVID-19 and cancer: lessons from a pooled meta-analysis. JCO Glob Oncol. 2020;6:557–9. PubMed https://doi.org/10.1200/GO.20.00097

63. Patterson TF, Thompson GR III, Denning DW, Fishman JA, Hadley S, Herbrecht R, et al. Practice guidelines for the diagnosis and management of aspergillosis: 2016 update by the Infectious Diseases Society of America. Clin Infect Dis. 2016;63:e1–60. PubMed https://doi.org/10.1093/cid/ciw326

64. Wahidi MM, Lamb C, Murgu S, Musani A, Shojaee S, Sachdeva A, et al. American Association for Bronchology and Interventional Pulmonology (AABIP) statement on the use of bronchoscopy and respiratory specimen collection in patients with suspected or confirmed COVID-19 infection. J Bronchology Interv Pulmonol. 2020;27:e52–4. PubMed https://doi.org/10.1097/LBR.0000000000000681

65. Koehler P, Cornely OA, Kochanek M. Bronchoscopy safety precautions for diagnosing COVID-19 associated pulmonary aspergillosis—a simulation study. Mycoses. 2020;64:55–9. PubMed

66. Verweij PE, Ananda-Rajah M, Andes D, Arendrup MC, Brüggemann RJ, Chowdhary A, et al. International expert opinion on the management of infection caused by azole-resistant *Aspergillus fumigatus*. Drug Resist Updat. 2015;21–22:30–40. PubMed https://doi.org/10.1016/j.drup.2015.08.001

67. Koehler P, Hamprecht A, Bader O, Bekeredjian-Ding I, Buchheidt D, Doelken G, et al. Epidemiology of invasive aspergillosis and azole resistance in patients with acute leukaemia: the SEPIA Study. Int J Antimicrob Agents. 2017;49:218–23. PubMed https://doi.org/10.1016/j.ijantimicag.2016.10.019

68. Vanderbeke L, Spriet I, Breynaert C, Rijnders BJA, Verweij PE, Wauters J. Invasive pulmonary aspergillosis complicating severe influenza: epidemiology, diagnosis and treatment. Curr Opin Infect Dis. 2018;31:471–80. PubMed https://doi.org/10.1097/QCO.0000000000000504

Appendix Table 1. Data sources describing patients with coronavirus disease–associated pulmonary aspergillosis, March–August 2020

Location and reference
Argentina
N.B. Fernández, et al. (*26*)
Australia
A. Sharma, et al. (*27*)
Austria
J. Prattes, et al. (*28*)
Belgium
L. Rutsaert, et al. (*29*)
C. Sarrazyn, et al. (*30*)
Brazil
D.R.Q. Lemos, et al. (*31*)
Denmark
M. Helleberg, et al. (*32*)
France
A. Alanio, et al. (*33*)
M. Blaize, et al. (*34*)
D. Dupont, et al. (*35*)*
J.P. Gangneux, et al. (*36*)
T. Ghelfenstein-Ferreira, et al. (*37*)
F.X. Lescure, et al. (*38*)
F. Schein, et al. (*39*)†
Germany
P. Koehler, et al. (*40*)
T. Lahmer, et al. (*41*)
Ireland
A. Mohamed, et al. (*42*)
Italy
S. Antinori S, et al. (*43*)
M. Bartoletti, et al. (*44*)
G. Bruno, et al. (*45*)
Netherlands
E.F.J. Meijer, et al. (*46*)
A.L.E. van Arkel, et al. (*47*)
S. Van Biesen, et al. (*48*)
Pakistan
N. Nasir, et al. (*49*)
Qatar
S. Abdalla, et al. (*50*)
Spain
I. Falces-Romero I, et al. (Appendix Reference *51*)†
M. Machado, et al. (Appendix Reference *52*)*
K.A. Marr KA, et al. (Appendix Reference *53*)*‡
Switzerland
F. Lamoth, et al. (Appendix Reference *54*)
United Kingdom
P.L. White, et al. (Appendix Reference *55*)

*The patients described in references *35*, Appendix Refence *52*, and Appendix Reference *53* were also documented in the FungiScope registry during the preparation of the current work.
†References *39* and Appendix Reference *51* describe patients that were not previously admitted to intensive care units.
‡Appendix Reference *53* includes cases from the United States.

Appendix Table 2. Data collection tool used in study on CAPA, March–August 2020*

Case published in the literature? *(If yes, please provide the DOI number)*:
Sex:
Age:
Country:
Year of infection:
Baseline conditions:
COVID-19:
- Length of treatment, *in days*:
- Treatment sequence:

ICU stay, *(yes/no)*:
- Length from ICU admission to CAPA, *in days*:
- ARDS, *(yes/no)*:
- Mechanical ventilation, *(yes/no)*:
    - o Start of ventilation before CAPA, *in days*:

Other relevant underlying conditions, (*please provide details*):
Diagnostics:
Microbiological evidence:
- Tests:
    - o Culture, *origin*:
    - o Microscopy, *origin*:
    - o Histology, *origin*:
    - o PCR, *origin*:
    - o Galactomannan, *origin and ODI*:
- Pathogen:
- Coinfection *(bacterial, fungal, viral)*:

Image abnormalities, *(yes/no)*:
- CT scan, (*please provide details*):
- x-ray, (*please provide details*):

Site(s) of CAPA involvement:
Antifungal susceptibility test:
Method:
MIC value per antifungal:
Antifungal treatment:
Length of treatment, *in days*:
Treatment sequence:
Outcome *(alive/dead):*
Observation days from CAPA diagnosis, *in days*:
Day of death:
Autopsy, *(yes/no)*:
Death attributable to, (*please provide details*):

*ARDS, acute respiratory distress syndrome; CAPA, COVID-19–associated pulmonary aspergillosis; COVID-19, coronavirus disease; CT, computed tomography scan; DOI, digital object identifier; ICU, intensive care unit; MIC, minimum inhibitory concentration; ODI, optical density index.

Appendix Table 3. Coronavirus disease treatment in patients with aspergillosis, March–August 2020

Treatment	No. (%)
Hydroxy-/chloroquine	110 (59.1)
Hydroxychloroquine	98 (52.7)
Chloroquine	12 (6.5)
Antiviral treatment	67 (36.0)
Lopinavir/ritonavir	56 (30.1)
Remdesivir	3 (1.6)
Lopinavir/ritonavir + remdesivir	2 (1.1)
Atazanavir	1 (0.5)
Darunavir + cobicistat	1 (0.5)
Lopinavir/ritonavir + darunavir	1 (0.5)
Lopinavir/ritonavir + oseltamivir	1 (0.5)
Lopinavir/ritonavir + ribavirin	1 (0.5)
Oseltamivir	1 (0.5)
Corticosteroids	69 (37.1)
Methylprednisolone	27 (14.5)
Hydrocortisone	9 (4.8)
Dexamethasone	9 (4.8)
Hydrocortisone + methylprednisolone	4 (2.2)
Dexamethasone + hydrocortisone + methylprednisolone	1 (0.5)
Dexamethasone + hydrocortisone + prednisolone	1 (0.5)
Dexamethasone + methylprednisolone	1 (0.5)
Methotrexate	1 (0.5)
Methylprednisolone + prednisone	1 (0.5)
Prednisolone	1 (0.5)
Prednisone	1 (0.5)
Nonspecified corticosteroids	13 (7.0)
Tocilizumab	51 (27.4)
Total	134 (72.0)

Appendix Table 4. Minimum inhibitory concentration values for *Aspergillus fumigatus* from 32 patients with coronavirus disease, March–August 2020

	Antifungal susceptibility testing, no. (IQR)†		
Antimicrobial drug, no. samples*	EUCAST n = 20	CLSI n = 1	E-test n = 11
Amphotericin B, 30	0.25 (0.25–0.5)	0.5 (0.5–0.5)	0.25 (0.125–0.38)
Anidulafungin, 5	0.008 (0.008–0.008)	0.008 (0.008–0.008)	
Caspofungin, 10	0.125 (0.125–0.125)		0.125 (0.125–0.25)
Micafungin, 1		0.008 (0.008–0.008)	
Isavuconazole, 22	0.5 (0.5–1)		0.06 (0.03–0.125)
Itraconazole, 25	0.5 (0.25–0.5)	16 (16–16)	0.38 (0.25–0.38)
Posaconazole, 24	0.064 (0.032–0.064)	0.5 (0.5–0.5)	0.125 (0.06–0.25)
Voriconazole, 31	0.5 (0.125–0.5)	2 (2–2)	0.094 (0.094–0.125)

*No. samples tested for susceptibility to each antifungal drug. One sample per patient.
†CLSI, Clinical and Laboratory Standards Institute (Reference *59* in Appendix); EUCAST, European Committee on Antimicrobial Susceptibility Testing (Reference *58* in Appendix).

Appendix Table 5. Antifungal treatments for aspergillosis in patients with coronavirus disease, March–August 2020

Treatment	no. (%)
VRZ	65 (34.9)
Isavuconazole	9 (4.8)
Liposomal AMB	8 (4.3)
VRZ + AMB + nebulized AMB, VRZ	7 (3.8)
Caspofungin	5 (2.7)
VRZ, isavuconazole	4 (2.2)
Posaconazole	3 (1.6)
VRZ, liposomal AMB	3 (0.6)
Anidulafungin	2 (1.1)
Caspofungin + VRZ, VRZ	2 (1.1)
Caspofungin, VRZ	2 (1.1)
Deoxycholate AMB	2 (1.1)
VRZ + AMB + nebulized AMB	2 (1.1)
Anidulafungin, anidulafungin + liposomal AMB, liposomal AMB	1 (0.5)
Anidulafungin, anidulafungin + liposomal AMB, liposomal AMB, anidulafungin + isavuconazole	1 (0.5)
Caspofungin + VRZ, VRZ, liposomal AMB	1 (0.5)
Caspofungin, isavuconazole	1 (0.5)
FLZ, VRZ	1 (0.5)
Isavuconazole + anidulafungin, anidulafungin	1 (0.5)
Isavuconazole + anidulafungin, isavuconazole	1 (0.5)
Isavuconazole, isavuconazole + nebulized AMB	1 (0.5)
isavuconazole, AMB lipid complex	1 (0.5)
liposomal AMB + anidulafungin	1 (0.5)
liposomal AMB, isavuconazole	1 (0.5)
liposomal AMB, isavuconazole, liposomal AMB	1 (0.5)
liposomal AMB, VRZ	1 (0.5)
liposomal AMB, VRZ, VRZ + caspofungin	1 (0.5)
AMB lipid complex	1 (0.5)
Micafungin, VRZ, isavuconazole, liposomal AMB	1 (0.5)
VRZ + anidulafungin	1 (0.5)
VRZ, ibrexafungerp*	1 (0.5)
VRZ, liposomal AMB + anidulafungin	1 (0.5)
VRZ, liposomal AMB, isavuconazole	1 (0.5)
VRZ, posaconazole	1 (0.5)
VRZ, VRZ + anidulafungin, liposomal AMB+ anidulafungin	1 (0.5)
VRZ, VRZ + caspofungin, VRZ	1 (0.5)
No antifungal treatment	49 (26.3)
Total	186 (100.0)

*Patient enrolled in the FURI Study (NCT 03059992; EuDra-CT 2017–000381–29). AMB, amphotericin B; FLZ, fluconazole; VRZ, voriconazole. + indicates drugs used in combination.

Appendix Table 6. Additional pathogens isolated from 186 patients with coronavirus disease–associated pulmonary aspergillosis, March–August 2020*

Sample	Pathogens	no. (%)
Blood	*Clostridium perfringens*	1 (0.5)
	Coagulase-negative Staphylococci	1 (0.5)
	Enterococcus spp.	1 (0.5)
	Enterococcus faecium	1 (0.5)
	Pseudomonas aeruginosa	2 (1.1)
	Staphylococcus aureus	1 (0.5)
	Staphylococcus epidermidis	3 (1.6)
	Staphylococcus epidermidis, Staphylococcus haemolyticus	1 (0.5)
	Stenotrophomonas maltophilia	1 (0.5)
	Unknown bacteria	1 (0.5)
Blood + catheter tip	Coagulase-negative Staphylococci, blood; *Klebsiella pneumoniae*, catheter tip	1 (0.5)
Blood + lung	*Enterobacter cloacae*, blood; *Pseudomonas aeruginosa*, NBL	1 (0.5)
	Enterococcus faecalis, blood and tracheal aspirate; *Staphylococcus capitis*, blood	1 (0.5)
	Enterococcus spp., blood; *Serratia* spp., NBL	1 (0.5)
	Pseudomonas aeruginosa, bronchial aspirate	1 (0.5)
	Staphylococcus aureus, blood + *Klebsiella* spp., NBL + *Haemophilus* spp., NBL	1 (0.5)
Blood + lung + deep soft tissue	*Enterococcus faecium*; *Elizabethkingia micirola*, bronchial aspirate	1 (0.5)
Blood + urine	*Facklamia hominis*, blood; *Escherichia coli*, urine	1 (0.5)
Central nervous system	Herpes simplex virus	1 (0.5)
Lung	*Acinetobacter baumannii*, tracheal aspirate	1 (0.5)
	Acinetobacter spp., tracheal aspirate (n = 1) and sputum (n = 1)	2 (1.1)
	Candida albicans, low-respiratory tract NOS	2 (1.1)
	Candida spp., sputum	1 (0.5)
	Citrobacter spp., *Pseudomonas aeruginosa*, and *Herpes simplex virus, bronchial aspirate*	1 (0.5)
	Corynebacterium striatum; Bordetella bronchiseptica, bronchial aspirate	1 (0.5)
	Klebsiella pneumoniae; *Staphylococcus aureus*, tracheal aspirate	1 (0.5)
	Klebsiella spp., NBL	1 (0.5)
	Klebsiella varicola; Candida albicans, tracheal aspirate	1 (0.5)
	Pseudomonas aeruginosa, tracheal aspirate	2 (1.1)
	Stenotrophomonas maltophilia, tracheal aspirate (n = 1) and sputum (n = 1)	2 (1.1)
	Unspecified yeasts, bronchial aspirate	1 (0.5)
Urinary tract	*Citrobacter amalonaticus*	1 (0.5)
	Enterococcus faecium	1 (0.5)
Total		40 (21.5)

*NBL, nondirected bronchial lavage; NOS, not otherwise specified

Appendix Table 7. Other conditions and exposures of 186 patients with coronavirus disease–associated pulmonary aspergillosis, March–August 2020

Condition	no. (%)
Acute renal failure requiring dialysis	13 (7.0)
Sepsis	10 (5.4)
Acute liver failure	9 (4.8)
Lymphopenia	8 (4.3)
Hypothyroidism	7 (3.8)
Rheumatic or autoimmune disorder	7 (3.8)
Ex-smoker	6 (3.2)
Smoker	6 (3.2)
Thrombocytopenia	6 (3.2)
Septic shock	4 (2.2)
Benign prostatic hypertrophy	3 (1.6)
Chronic liver failure	3 (1.6)
Non-ST segment elevation myocardial infarction	3 (1.6)
Acute renal failure requiring renal substitution	2 (1.1)
Chronic renal failure requiring dialysis	2 (1.1)
Depression	2 (1.1)
Exposure to fungicides or manipulation of organic matter possibly containing triazole-resistant isolates of *Aspergillus fumigatus*	2 (1.1)
History of pulmonary tuberculosis	2 (1.1)
Multiorgan dysfunction syndrome	2 (1.1)
Liver failure not otherwise specified	2 (1.1)
Psychiatric disorder	2 (1.1)
Alcoholism	1 (0.5)
Aneurysm coiling right arteria vertebralis	1 (0.5)
Atrial myxoma	1 (0.5)
CD8+ T-cell lymphocytosis	1 (0.5)
Contact with a patient with coronavirus disease	1 (0.5)
Cured thyroid cancer	1 (0.5)
Dementia	1 (0.5)
Epilepsy	1 (0.5)
Flavectomy and nucleotomy	1 (0.5)
Gas gangrene	1 (0.5)
Glaucoma	1 (0.5)
Hospital-acquired pneumonia	1 (0.5)
Hypercholesterinemia	1 (0.5)
Hyperkalemia	1 (0.5)
Hyperparathyroidism secondary to chronic kidney disease	1 (0.5)
Hyperthyroidism	1 (0.5)
Hypercholesterolemia	1 (0.5)
Hyperlipidemia	1 (0.5)
Laparoscopic cholecystectomy for cholecystitis	1 (0.5)
Mastoiditis	1 (0.5)
Osteopenia	1 (0.5)
Polyneuropathy	1 (0.5)
Posterior reversible encephalopathy syndrome	1 (0.5)
Previous hepatitis B	1 (0.5)
Reactivation of cytomegalovirus	1 (0.5)
Recent stroke	1 (0.5)
Sinusitis	1 (0.5)
Sleep apnea syndrome	1 (0.5)
Stenosis of the left internal carotid artery	1 (0.5)
Suspected pulmonary fibrosis	1 (0.5)
Thyroidectomy	1 (0.5)
Umbilical hernia	1 (0.5)
Vertebral disc herniation left L4/5	1 (0.5)
Total	67 (36.0)

Appendix Table 8. Distribution of patients with coronavirus disease–associated pulmonary aspergillosis, March–August 2020

Country	Data source, no. (%)			Total, no. (%)
	FungiScope	FungiScope + Literature	Literature	
Argentina		1 (0.5)		1 (0.5)
Australia			1 (0.5)	1 (0.5)
Austria			1 (0.5)	1 (0.5)
Belgium	4 (2.2)		4 (2.2)	8 (4.3)
Brazil	2 (1.1)		1 (0.5)	3 (1.6)
Denmark			2 (1.1)	2 (1.1)
France	18 (9.7)	9 (4.8)	12 (6.5)	39 (21.0)
Germany	17 (9.1)	4 (2.2)	2 (1.1)	23 (12.4)
Ireland			1 (0.5)	1 (0.5)
Italy	4 (2.2)	30 (16.1)	2 (1.1)	36 (19.4)
Mexico	6 (3.2)			6 (3.2)
Netherlands	3 (1.6)		11 (5.9)	14 (7.5)
Pakistan			9 (4.8)	9 (4.8)
Qatar			2 (1.1)	2 (1.1)
Spain	3 (1.6)	23 (12.4)		26 (14.0)
Switzerland			3 (1.6)	3 (1.6)
United Kingdom			11 (5.9)	11 (5.9)
Total	57 (30.6)	67 (36.0)	62 (33.3)	186 (100.0)
